# Transforming Waste into Valuable Resources: Mo_2_C Nanoparticles Modified Waste Pinecone-Derived Carbon as an Effective Sulfur Host for Lithium–Sulfur Batteries

**DOI:** 10.3390/ma18051141

**Published:** 2025-03-04

**Authors:** Zhe Yang, Yicheng Han, Kai Chen, Guodong Zhang, Shuangxi Xing

**Affiliations:** 1 Faculty of Chemistry, Northeast Normal University, Changchun 130024, China; yangzhe688@nenu.edu.cn (Z.Y.); hanyicheng333@nenu.edu.cn (Y.H.); 2 Fujian Provincial Key Laboratory for Soft Functional Materials, Department of Physics, Research Institute for Biomimetics and Soft Matter, Xiamen University, Xiamen 361005, China; 19820210156894@stu.xmu.edu.cn

**Keywords:** pinecone-derived carbon, Mo_2_C nanoparticles, adsorption and catalysis, sulfur host, Li-S batteries

## Abstract

In this paper, the natural waste pinecone as a carbon precursor for the generation of satisfactory sulfur host materials in lithium–sulfur batteries was realized by introducing molybdenum carbide nanoparticles into the derived carbon structure. The conductive pinecone-derived carbon doped with N, O reveals an expansive specific surface area, facilitating the accommodation of a higher sulfur load. Moreover, the integration of Mo_2_C nanoparticles also significantly enhances its chemical affinity and catalytic capacity for polysulfides (LiPSs) to alleviate the shuttle effect and accelerate sulfur redox conversion. As a result, the WPC-Mo_2_C/S electrode displays excellent electrochemical performance, including a low capacity decay rate of 0.074% per cycle during 600 cycles at 1 C and an outstanding rate capacity (631.2 mAh g^−1^ at 3 C). Moreover, with a high sulfur loading of 5.5 mg cm^−2^, the WPC-Mo_2_C/S electrode shows a high area capacity of 5.1 mAh cm^−2^ after 60 cycles at 0.2 C.

## 1. Introduction

Lithium–sulfur batteries are regarded as most promising energy storage devices due to their excellent specific capacity (1675 mAh g^−1^) and energy density (2600 Wh kg^−1^) [1,2,3]. Meanwhile, because of their low cost and non-pollution, they have great prospects in terms of their application [4]. However, the sulfur cathode still faces the following problems: (1) The low conductivity of sulfur and its discharge products (Li_2_S/Li_2_S_2_) affect the utilization of active sulfur. (2) The shuttle effect and slow conversion kinetics of LiPSs result in a low Coulomb efficiency and loss of capacity. (3) The volume expansion of the sulfur cathode destroys the structure of the cathode, decreasing its stability [5,6,7]. The above problems have seriously affected the application and further development of lithium–sulfur batteries [8,9].

To address the above problems, conductive carbon materials with high electronic conductivity are commonly used as sulfur carriers, owing to their role in electron transport in lithium–sulfur batteries [10,11,12]. In addition, their large specific surface area and porous structure provide sufficient space for the high sulfur loading and volume expansion of the cathode, improving the stability of the cathode [13]. Moreover, carbon materials can be strategically doped with N and P elements to achieve the effective adsorption of LiPSs [14]. Furthermore, on the other hand, polar materials, such as Mo_2_C, CoP, and Ni_12_P_5_, are also widely used as sulfur hosts through polar interaction with LiPSs, which can achieve the effective adsorption of LiPSs, inhibit the shuttle effect, and catalyze to promote sulfur redox conversion, thereby improve the capacity stability of Li-S batteries [15,16,17,18].

Among all the carbon materials, biomass-derived carbon is much more promising because of its outstanding advantages, including strong physical and chemical adsorption, rich sources, low cost, and environmental friendliness, which makes it a satisfactory host material in Li-S batteries [19,20]. Up to now, many kinds of biomass materials have been explored for a wide range of applications in energy conversion [21,22]. Wang et al. obtained a monolith of porous carbon derived from rice husk via zinc-assisted pyrolysis, where the conductive network improves the conductance and buffers the volume change. The porous carbon exhibited a high surface area and rich pores, and excellent electrochemical performance has been obtained thereby [23]. In addition, Sharma investigated the use of porous carbon derived from tamarind peel biomass as a sulfur host. Tamarind peel underwent sequential pyrolysis, activation, and nitrogen doping to yield diverse porous carbon samples. The hierarchical porous structure and polar surfaces introduced by nitrogen doping improve sulfur utilization and reduce polysulfide shuttling, exhibiting notable electrochemical performance in Li–S batteries [24]. The above materials demonstrate the following advantages: (1) high electrical conductivity, (2) an excellent specific surface area and porous structure, and (3) non-metal elements doping, such as with N and P, which changes the characteristics and structure of the carbon materials and realizes the adsorption of LiPSs [25]. Pinecone is a kind of natural material that is widely distributed around the northeast part of China. It has been utilized as an active component in supercapacitors; however, to our best knowledge, it has not been widely used as the sulfur host material in Li-S batteries [26,27], which may originate from its poor adsorption of LiPSs, because of which serious capacity decay occurs.

In this work, waste pinecone-derived carbon modified by Mo_2_C (WPC-Mo_2_C) was obtained through the activation and carbonization process, where WPC provides a satisfactory specific surface area for the loading of active materials, and Mo_2_C enhances the adsorption and catalysis of LiPSs, inhibiting the shuttle effect of LiPSs and accelerating the sulfur redox conversion. Thanks to the synergistic effect between the two materials, the WPC-Mo_2_C/S cathode demonstrates improved conductivity, reduced volume expansion, and improved structure stability. As a result, the WPC-Mo_2_C/S electrode exhibits a minimal capacity decay rate of 0.074% after 600 cycles at 1 C and a high capacity of 631.2 mAh g^−1^ at 3 C. Moreover, the WPC-Mo_2_C/S electrode shows an excellent area capacity of 5.1 mAh cm^−2^ after 60 cycles when the sulfur loading capacity reaches 5.5 mg cm^−2^, exceeding the standards for commercial applications (4 mAh cm^−2^).

## 2. Materials and Methods

### 2.1. Materials

Waste pinecones were collected from natural sources. Analytical-grade solvent (ethylene glycol) and metal salts ((NH_4_)_6_Mo_7_O_24_·4H_2_O, S, and LiNO_3_) were obtained from Aladdin Scientific Corp. (Riverside, CA, USA). Other chemicals, including acetone, DME, DOL, Li_2_S, and LiTFSI with 100%, 99.9%, 99.8%, 99.5%, and 98% purities, were acquired from Sigma-Aldrich (St. Louis, MO, USA). Battery-level LA133 and Ketjen Black (KB) were employed as binder and conductivity enhancers on a carbon cloth substrate (WOS 1009) and were purchased from Canrd Co., Ltd. (Xiamen, China). Similarly, battery-level lithium foil (anode) and Polyvinylidene Fluoride (PVDF) were obtained from Shenzhen Kejing Co., Ltd. (Shenzhen, China).

### 2.2. Characterization

Transmission electron microscopy (TEM, JEM-2100F, Tokyo, Japan) and scanning electron microscopy (SEM, JSM-IT300, Tokyo, Japan) were employed to investigate the morphology of the samples. X-ray powder diffraction (XRD) was recorded on a Siemens D5005 diffractometer (Munich, Germany) using Cu-K_α_ radiation (*λ* = 1.5418 Å, 30 mA, 40 kV). X-ray photoelectron spectroscopy (XPS) spectra were observed on an ESCALAB 250 (Waltham, MA, USA) using monochromatic Al K_α_ excitation (1486.6 eV). Raman spectra were observed using a LabRAM XploRA laser Raman spectrometer (HORIBA, Kyoto, Japan) from 800 to 2000 cm^−1^. The Brunauer–Emmett–Teller (BET) specific surface area was determined using a Micromeritics ASAP 2020 nitrogen adsorption instrument (Norcross, GA, USA). The thermogravimetric analysis (TGA) was carried out on a Shimadzu DTG-60H analyzer (Kyoto, Japan) heated from room temperature to 600 °C at a ramp rate of 5 °C min^−1^ under N_2_.

### 2.3. The Pretreatment and Activation of Pinecone

The cleaning of the pinecones was the first step, and deionized water (DIW) was used to remove dirt and impurities from the waste pinecone. Then, an appropriate amount of pinecone was crushed into powder in a grinder, and then the powder was dried at 60 °C for 24 h. After that, the power was heated to 600 °C at a rate of 5 °C min^−1^ and maintained for 2 h under a continuous flow of nitrogen to obtain the pinecone-derived carbon preliminarily (p-WPC).

The second step is the activation of p-WPC with KOH. The p-WPC was put into a beaker with 20 wt% KOH, stirred for 2 h, and then dried at 60 °C for 24 h. After that, the p-WPC was heated to 900 °C at a rate of 5 °C min^−1^ and maintained for 2 h. Finally, the activated p-WPC was washed with 2 M HCl several times until the solution was neutral, and the product was named WPC.

### 2.4. Synthesis of WPC-Mo_2_C and WPC

The activated sample was mixed with (NH_4_)_6_Mo_7_O_24_·4H_2_O at a 3:1 mass ratio, followed by calcination (900 °C) at a rate of 5 °C min^−1^, and maintained for 2 h under an inert atmosphere (N_2_). Finally, the resultant sample was labeled as WPC-Mo_2_C. For reference, pure WPC was attained under similar conditions, except the metal salt ((NH_4_)_6_Mo_7_O_24_·4H_2_O) was excluded. The preparation process of WPC-Mo_2_C can be described by the following formulas:

(NH_4_)_2_MoO_4_ = MoO_3_ + 2NH_3_ + H_2_O (1)

2MoO_3_ + 3C = 2Mo + 3CO_2_ (2)

2Mo + C = Mo_2_C (3)

### 2.5. Synthesis of WPC-Mo_2_C/S and WPC/S

A classical melting diffusion method was employed to load sulfur in the synthesized host material. The sulfur and the host material were fully ground in an agate mortar with a 7:3 mass ratio for 1 h, and the color of the resultant mixture turned black. Then, this black-colored mixture was placed into a glass vial without tightening the vial, which was followed by heating in a muffle furnace at 155 °C for 12 h. To ensure the high loading of sulfur, the above-mentioned process was repeated five times. The same process was employed with WPC to make the WPC/S sample.

### 2.6. Preparation of PC-Mo_2_C/S Electrode

A mixture of WPC-Mo_2_C/S, KB, and PVDF with a mass ratio of 7:2:1 was ground to form a homogeneous slurry of the WPC-Mo_2_C/S electrode. The slurry was stirred for 12 h at room temperature, and then the doctor blade method was used to uniformly coat it onto carbon-coated aluminum foil. The coated foil was dried at 60 °C overnight under vacuum conditions. Subsequently, the dried aluminum foil with the WPC-Mo_2_C/S sample was cut into 12 mm diameter disks. The sample of the WPC/S electrodes was synthesized in the same way as above.

### 2.7. Electrochemical Measurements

An Ar-filled glove box (O_2_, H_2_O < 0.1 ppm) was used to assemble CR2025 coin cells. The LAND CT2001A battery test system was employed to test the battery performance. An amount of 1.0 M LiTFSI with 0.2 M LiNO_3_ in DOL/DME (1:1) was used as an electrolyte, and a 1.7–2.8 V voltage window was used to record the galvanostatic charge/discharge cycles at ambient temperature (25 °C). The mass loading of sulfur is 1.2 mg cm^−2^. Electrolyte/sulfur ratios of 25 μL mg^−1^ and 15 μL mg^−1^ were used for the normal and high sulfur loading of the 5.5 mg cm^−2^ cells, respectively. The galvanostatic intermittent titration technique (GITT) analysis was carried out at 0.1 C, with a charging/discharging time of 15 min, and then the battery was stationed for 30 min. A CHI660E workstation was used to record the CV measurements, and the scan rate varied from 0.1 to 0.5 mV s^−1^.

### 2.8. Lithium Sulfide Adsorption Test

A mixture of DOL and DME (1:1 by volume) was used to dissolve lithium sulfide and sulfur with a molar ratio of 1:5 to make an Li_2_S_6_ solution, which was stirred at 80 °C in an Ar-filled glove box. Then, a fixed mass (10 mg) of the active material (WPC-Mo_2_C or WPC) was added into a 5 mM Li_2_S_6_ (2 mL) solution. Then, the resulting solution with the active material was aged several hours to assess the adsorption properties of these materials.

### 2.9. Symmetrical Cell Assembly and Measurements

Both the working and counter electrodes in the symmetrical cells were made up of either as-prepared WPC or WPC-Mo_2_C, and a specific amount of the 0.5 M Li_2_S_6_ electrolyte was used to make the symmetrical cells. The assembled symmetrical cells were subjected to the CV tests at a scan rate of 0.05 V s^−1^. The potential window of the CV scans was from −1 to 1 V.

### 2.10. Li_2_S Nucleation and Dissolution Experiments

The carbon paper was loaded with a host material (1 mg), and the lithium metal worked as a cathode and anode. Amounts of 10 μL of 0.25 M Li_2_S_8_ and 10 μL of LiTFSI catholyte and anolyte were added to the cathode and anode sides. After discharging the cells to 2.06 at a low current of 0.112 mA, the potential was maintained at 2.05 V to facilitate Li_2_S nucleation until the current reached below 10^−5^ A. In addition, the same process was repeated at 1.70 V, and then the potential was maintained at 2.30 V to facilitate Li2S dissolution until the current dropped below 10^−5^ A. Finally, the Li_2_S nucleation and dissolution capacity were calculated based on Faraday’s law.

## 3. Results and Discussions

### 3.1. Results

**Scheme 1:**
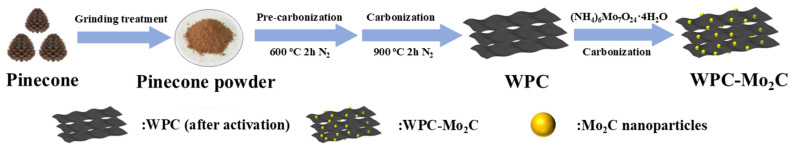
Illustration of the synthetic process of WPC-Mo_2_C.

The synthesis process for waste pinecone-derived carbon decorated with Mo_2_C nanoparticles (WPC-Mo_2_C) is illustrated in Scheme 1. Firstly, the bulk pinecones were crushed into powder, followed by carbonization at 600 °C, and the pinecone-derived carbon was obtained preliminarily (p-WPC). Secondly, the p-WPC was activated with a 20 wt% KOH solution and carbonized at 900 °C, and then the activated carbon (WPC) was obtained after neutralization with 2 M HCl. After that, the WPC was mixed with (NH_4_)_6_Mo_7_O_24_·4H_2_O at 3:1 and then carbonized at 900 °C for 2 h to obtain a WPC-Mo_2_C composite.

**Figure 1:**
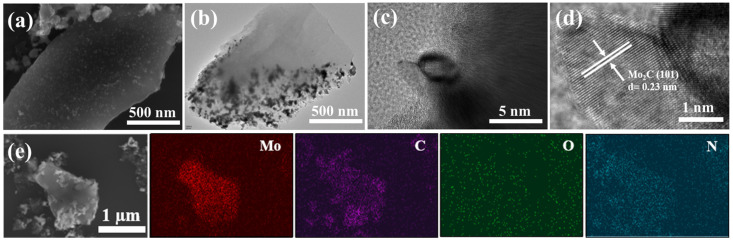
(**a**) SEM image of WPC-Mo_2_C; (**b**) TEM image of WPC-Mo_2_C; (**c**,**d**) HRTEM image of WPC-Mo_2_C; (**e**) EDS mapping with corresponding elements of Mo, C, O, and N of WPC-Mo_2_C.

The morphology of WPC-Mo_2_C was tested by SEM and TEM (Figure 1a,b), and the results show that abundant nanoparticles anchor on the surface of the WPC. To further confirm the structure of these nanoparticles, HRTEM tests were performed (Figure 1c,d), and a lattice distance of 0.23 nm is clearly observed in Figure 1d, which aligns with the (101) plane of Mo_2_C [28]. The EDS element mapping images (Figure 1e) illustrate the distribution of Mo, C, O, and N elements in the WPC-Mo_2_C composite material. In addition, the integration of elemental Mo and elemental C can also be obtained from the EDS images, confirming the existence of Mo_2_C nanoparticles in other ways. The specific surface area of WPC-Mo_2_C and WPC was tested as 1510.3 m^2^ g^−1^ and 971.6 m^2^ g^−1^ (Figures S1 and S2), respectively, and the difference in specific surface area reflects the effect of the introduction of Mo_2_C. Moreover, the process of KOH activation also produces new micropore characteristics at 1.98 nm in WPC-Mo_2_C.

**Figure 2:**
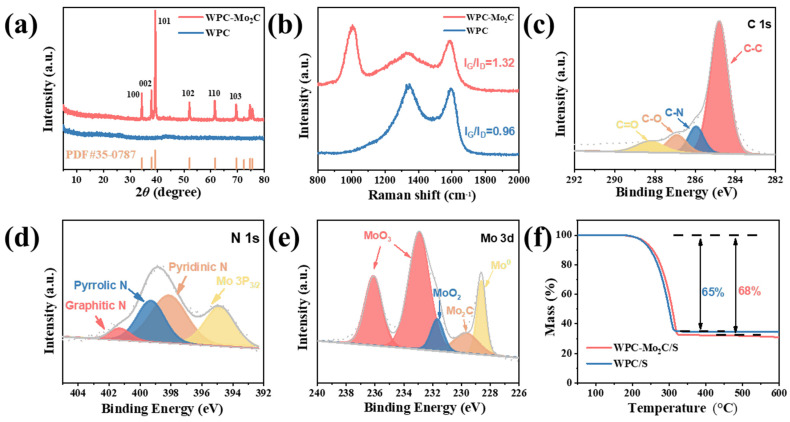
(**a**) XRD patterns of WPC-Mo_2_C and WPC; (**b**) Raman spectra of WPC-Mo_2_C and WPC; (**c**) C 1s, (**d**) N 1s, and (**e**) Mo 3d XPS spectra of WPC-Mo_2_C; (**f**) TGA curves of WPC-Mo_2_C/S and WPC/S.

To further analyze the structure of WPC-Mo_2_C and WPC, XRD was carried out, and the relevant results are shown in Figure 2a. The XRD pattern of WPC-Mo_2_C shows six diffraction peaks corresponding to the (100), (002), (101), (102), (110), and (103) planes of the Mo_2_C standard card (PDF#35-0787), respectively. No obvious peaks can be found in the XRD pattern of WPC, consistent with the general characteristics of carbon materials [29]. An ICP test was conducted to determine the content of the Mo element in WPC-Mo_2_C. According to the test result, the content of the Mo element is about 0.91 wt% (Table S1). The Raman spectra of WPC-Mo_2_C and WPC are shown in Figure 2b, and two peaks at 1350 and 1580 cm^−1^ of WPC-Mo_2_C and WPC, known as the D and G bands, respectively, can be observed. The intensity ratio of these two peaks (*I*_D_/*I*_G_) of WPC-Mo_2_C and WPC are 1.32 and 0.96, respectively, illustrating the higher degree of graphitized carbon in WPC-Mo_2_C, and this is beneficial for the faster transport of electron ions in Li-S batteries [30]. In addition, a peak at around 1000 cm^−1^ is detected in WPC-Mo_2_C, which belongs to characteristic peaks of Mo compounds [31]. To analyze the chemical composition of WPC-Mo_2_C, XPS tests were carried out, and the XPS full spectrum of WPC-Mo_2_C confirms the existence of Mo, C, O, and N elements (Figure S3). As shown in Figure 2c, the C 1s spectrum of WPC-Mo_2_C can be split into four peaks located at 284.8 eV (C–C), 285.9 eV (C–N), 286.9 eV (C–O), and 288.3 eV (C=O). The N 1 s spectrum (Figure 2d) displays four peaks, including Mo 3P_3/2_ (394.8 eV), pyridinic-N (398.1 eV), pyrrolic-N (399.3 eV), and graphitic-N (401.3 eV); the N element contains the peaks about Mo, which also correspond to the full spectrum of the XPS. Moreover, pyridinic-N and pyrrolic-N can help to form lithium–nitrogen bonds, and this contributes to suppress the shuttle effect and alleviate the dissolution of LiPSs. Graphitic-N helps to improve the conductivity of the WPC-Mo_2_C. In the Mo 3d spectrum (Figure 2e), the peaks at 228.6 eV and 229.6 eV correspond to Mo^0^ and Mo_2_C, respectively, while the peaks of MoO_2_ and MoO_3_ at 231.7 eV, 232.9 eV, and 236.1 eV are due to the oxidation caused by exposure to the air. The TGA curves of WPC-Mo_2_C/S and WPC/S are shown in Figure 2f. The WPC-Mo_2_C/S shows a sulfur content of 68%, while the WPC/S shows a sulfur content of 65%, which may be related to the larger surface area of WPC-Mo_2_C/S.

**Figure 3:**
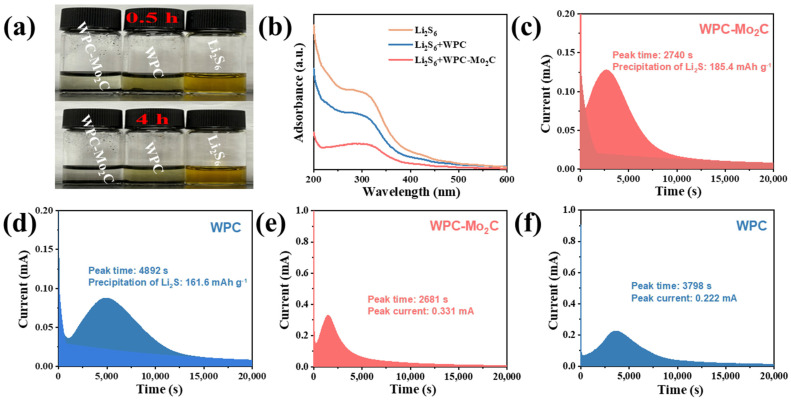
(**a**) Visual adsorption tests and (**b**) the corresponding UV–vis spectra of WPC-Mo_2_C-Li_2_S_6_, WPC-Li_2_S_6,_ and pure Li_2_S_6_. (**c**) Precipitation profiles of Li_2_S at 2.05 V with WPC-Mo_2_C and WPC (**d**). (**e**) Dissolution profiles of Li_2_S at 2.30 V with WPC-Mo_2_C and WPC (**f**).

In order to verify the adsorption capacity of WPC-Mo_2_C and WPC for LiPSs, an adsorption experiment of WPC-Mo_2_C and WPC for Li_2_S_6_ was carried out. As shown in Figure 3a, the yellow color in the WPC-Mo_2_C with Li_2_S_6_ faded at 0.5 h, then disappeared at 4 h, demonstrating the excellent adsorption capacity of WPC-Mo_2_C. In addition, a UV–vis adsorption test was conducted, and the corresponding results can be seen in Figure 3b. The results further confirm the strong adsorption capacity to LiPSs of WPC-Mo_2_C, which can inhibit the shuttle effect of LiPSs effectively. To further prove the catalytic capacity of LiPSs of WPC-Mo_2_C and WPC, the influence of WPC-Mo_2_C and WPC on the nucleation/dissolution process of Li_2_Ss was investigated. As shown in Figure 3c,d, the WPC-Mo_2_C exhibits an earlier current response and larger deposition capacity (2740 s/185.4 mAh g^−1^) than WPC (4892 s/161.6 mAh g^−1^). In the dissolution process of Li_2_Ss, the WPC-Mo_2_C exhibits an earlier current response and larger peak current (2681 s/0.331 mA) than WPC (3798 s/0.222 mA) in Figure 3e,f. The above results illustrate that the WPC-Mo_2_C can accelerate the deposition and dissolution of LiPSs, catalyzing and facilitating the liquid–solid reactions of the LiPSs’ process.

**Figure 4:**
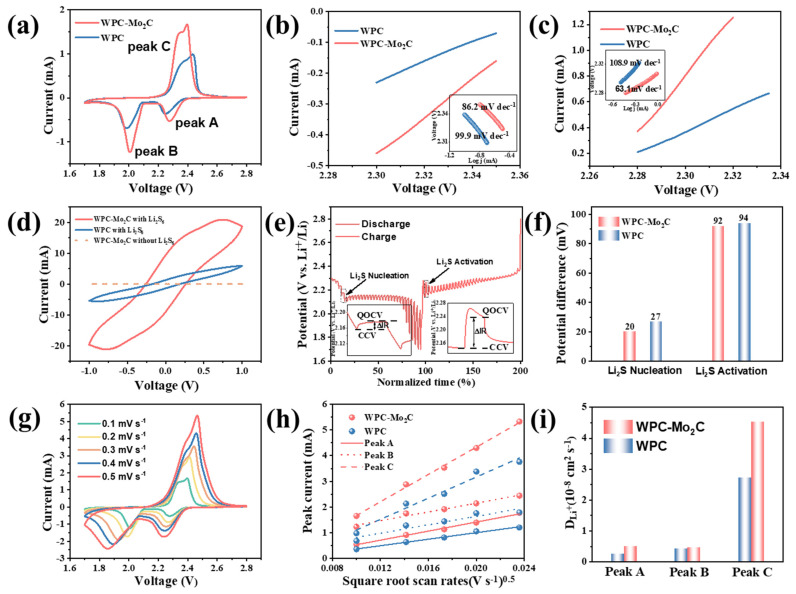
(**a**) CV curves of WPC-Mo_2_C and WPC at a scan rate of 0.1 mV s^−1^; an enlarged section of peak A and peak C of WPC-Mo_2_C (**b**) and WPC (**c**) and the corresponding Tafel plot (inset); (**d**) CV curves of symmetric cells at a scan rate of 50 mV s^−1^; (**e**) GITT profile of WPC-Mo_2_C and (**f**) the corresponding potential difference in quasi open-circuit voltage and closed-circuit voltage at the Li_2_S nucleation and Li_2_S activation points of WPC-Mo_2_C and WPC; (**g**) CV curves of WPC-Mo_2_C at a scan rate of 0.1–0.5 mV s^−1^; (**h**) the linear fits of the peak currents of WPC-Mo_2_C and WPC and (**i**) the corresponding Li^+^ diffusion coefficients at different peaks.

To further investigate the influence of different electrodes on the electrochemical performance of lithium–sulfur batteries, WPC-Mo_2_C/S and WPC/S were respectively used as cathodes and metal Li as anode electrodes. CV curves with different cathodes were measured at 0.1 mV s^−1^, as shown in Figure 4a. The two reduction peaks, A and B, correspond to the reduction from S_8_ to long-chain LiPSs (Li_2_S*_n_*, 4 ≤ *n* ≤ 8) and further to insoluble Li_2_S_2_/Li_2_S, and the oxidation peak C corresponds to the oxidation of the LiPSs to S_8_. The peaks A, B, and C correspond to currents of 0.52, 1.24, and 1.67mA for WPC-Mo_2_C materials. For WPC materials, peaks A, B, and C correspond to currents of 0.36, 0.68, and 0.98mA. The WPC-Mo_2_C/S electrode shows a smaller polarization and larger current response. In order to further verify the kinetic process, the curves at peaks A and C are selected, and the corresponding Tafel slopes are calculated, as shown in Figure 4b,c. The Tafel slope of the WPC-Mo_2_C/S electrode is 86.2 mV dec^−1^, lower than that of the WPC/S (99.9 mV dec^−1^) electrode at peak A. In addition, the Tafel slope of the WPC-Mo_2_C/S electrode is 63.1 mV dec^−1^, which is also lower than that of the WPC/S (108.9 mV dec^−1^) electrode at peak C. In Figure 4b,c, the curves of WPC-Mo_2_C both show a quicker decline trend, and the above results illustrate that the WPC-Mo_2_C/S electrode shows a faster transfer rate and an accelerated kinetic process in Li-S batteries. These results further indicate that the introduction of Mo_2_C can effectively improve the conductivity of the electrode and catalyze the conversion process of LiPSs. To demonstrate the catalytic effect of the host materials on LiPSs, the symmetric cells were respectively assembled with WPC-Mo_2_C and WPC electrodes, and the results are shown in Figure 4d. First of all, no obvious peak is observed in the CV curves of the cells without Li_2_S_6_; however, the cells with WPC-Mo_2_C show a wider profile, larger CV curve area, and larger response current intensity than WPC, indicating that WPC-Mo_2_C possess a stronger catalytic conversion ability for LiPSs than WPC. Moreover, GITT tests were conducted in order to explore the influence of host materials on the dynamic reaction in Li-S batteries, and the results are shown in Figure 4e and Figure S4. The potential difference between Li_2_S nucleation and activation points is shown in Figure 4f. For batteries assembled with the WPC-Mo_2_C electrode, the nucleation point potential difference is 20 mV, and the activation point potential difference is 92 mV, both lower than the batteries assembled with WPC electrode (27 and 94 mV), revealing its alleviated polarization and accelerated kinetics. Finally, to calculate the Li^+^ transfer rate in Li-S batteries, the CV curves with different cathodes at 0.1–0.5 mV s^−1^ were measured (Figure 4g and Figure S5); the WPC-Mo_2_C electrode corresponds to a higher peak at 0.1–0.5 mV s^−1^, and the linear fits of the peak currents of the WPC-Mo_2_C and WPC cathodes are shown in Figure 4h. According to the classical Randles–Sevcik equation, *I*_P_ = 2.69 × 10^5^ × *n*^1.5^A*D*_Li_^+0.5^*C*_Li_^+^ν^0.5^, the Li⁺ diffusion coefficients of the WPC-Mo_2_C electrode are calculated to be *D*_Li_^+^ (A) = 4.98 × 10^−9^ cm^2^ s^−1^, *D*_Li_^+^ (B) = 4.69 × 10^−9^ cm^2^ s^−1^, and *D*_Li_^+^ (C) = 4.52 × 10^−8^ cm^2^ s^−1^, higher than those of the WPC electrode (Figure 4i and Table S2).

**Figure 5:**
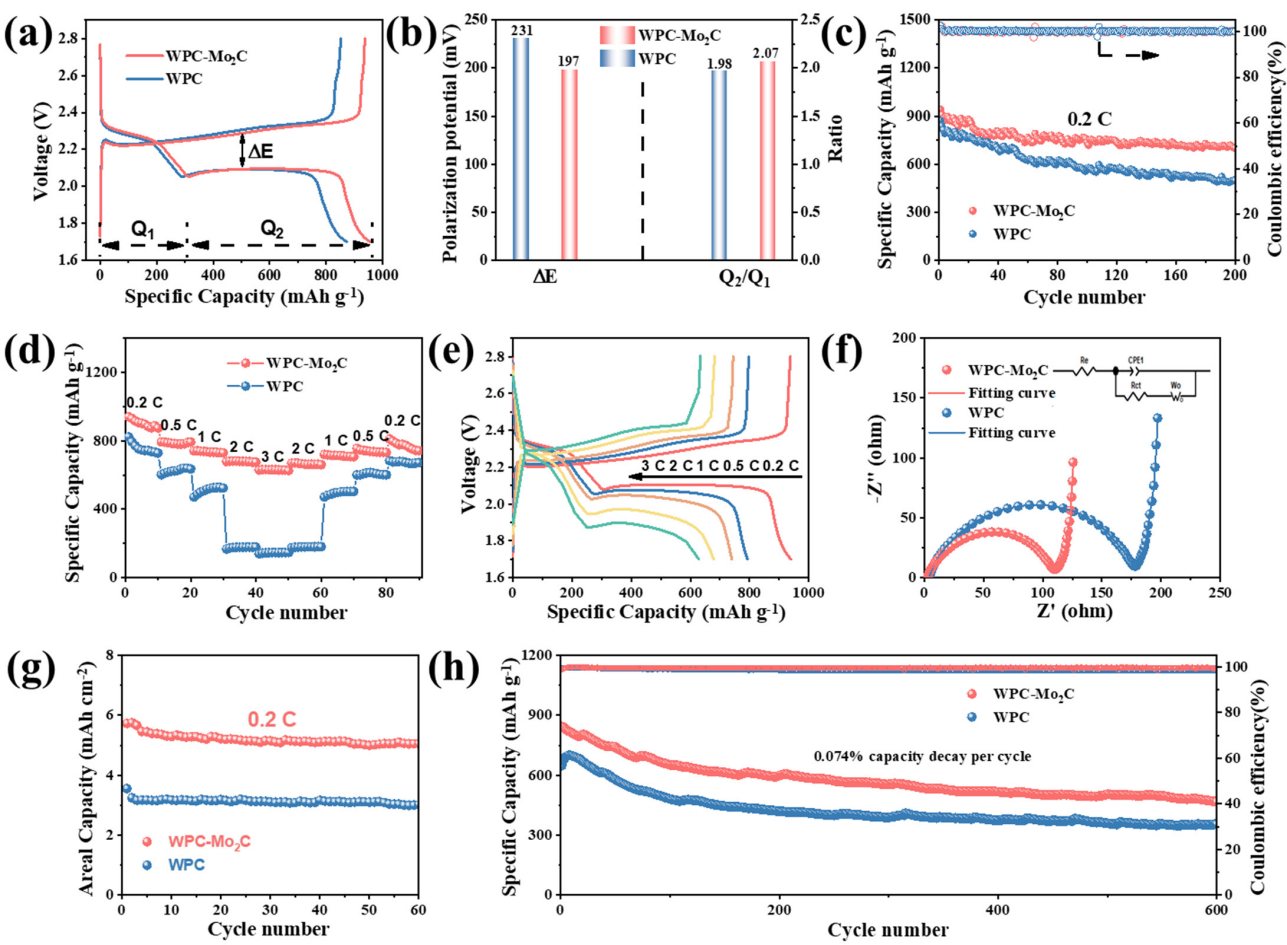
Electrochemical performance of WPC-Mo_2_C and WPC electrodes: (**a**) discharge and charge curves for the first cycle at 0.2 C; (**b**) ΔE and Q_2_/Q_1_ ratios from charge/discharge curves at 0.2 C; (**c**) cycling performance at 0.2 C; (**d**) rate performance at 0.2–3 C and (**e**) the corresponding charge/discharge curves at 0.2–3 C; (**f**) EIS profiles; (**g**) corresponding potential difference in quasi open-circuit voltage and closed-circuit voltage at the Li_2_S nucleation and Li_2_S activation points of WPC-Mo_2_C and WPC; (**g**) cycling performance at 0.2 C with a high sulfur loading; (**h**) cycling performance at 1 C.

The electrochemical performance of the WPC-Mo_2_C and WPC electrodes is shown in Figure 5. First, the discharge and charge curves for the first cycle at 0.2 C (Figure 5a) show two discharge platforms and one charge platform, which corresponds to the CV curves. In addition, compared with the cell with the WPC electrode, the cell with the WPC-Mo_2_C electrode shows less polarization (Δ*E* = 197 mV) and a larger Q_2_/Q_1_ ratio (2.07) in Figure 5b, and the results reflect the fast dynamic process and the high utilization of sulfur of the WPC-Mo_2_C electrode. As shown in Figure 5c, the WPC-Mo_2_C/S electrode presents a specific capacity of 942.2 mAh g^−1^ at 0.2 C, with a 0.12% capacity of decay per cycle after 200 cycles, better than that of the WPC/S electrode. In addition, the WPC-Mo_2_C/S electrode shows a specific capacity of 631.2 mAh g^−1^ at 3 C (Figure 5d); in contrast, the WPC electrode only shows a discharge capacity of 138.6 mAh g^−1^ at 3 C. The above results highlight the outstanding rate performance of the WPC-Mo_2_C/S electrode. Moreover, the charge and discharge curves of the WPC-Mo_2_C/S electrode at 0.2, 0.5, 1, 2, and 3 C are shown in Figure 5e, and two obvious discharge platforms at 3 C are observed, which verifies the excellent conductivity and stability of the WPC-Mo_2_C/S electrode. And it is apparent that the polarization between charge and discharge curves becomes obvious with the current increase. The EIS profiles of the cells with WPC-Mo_2_C/S and WPC/S electrodes are shown in Figure 5f. The cell with the WPC-Mo_2_C/S electrode exhibits a smaller interface resistance (*R_s_*) of 2.79 Ω and charge transfer resistance (*R_ct_*) of 110 Ω than the cell with the WPC/S electrode (4.73 Ω for *R_s_* and 178 Ω for *R_ct_*, summarized in Table S3). This illustrates that the WPC-Mo_2_C/S electrode accelerates the conversion of LiPSs and enhances the redox kinetics of the batteries. Meanwhile, the WPC-Mo_2_C/S electrode demonstrates an excellent area specific capacity of 5.1 mAh cm^−2^ after 60 cycles at 0.2 C with a sulfur loading of 5.5 mg cm^−2^ in Figure 5g, better than the standard for commercial lithium–sulfur batteries (4.0 mAh cm^−2^). Finally, the WPC-Mo_2_C/S electrode exhibits an excellent specific capacity of 843.3 mAh g^−1^ at 1 C and with a 0.074% capacity decay per cycle after 600 cycles. Moreover, the cell with the WPC-Mo_2_C/S electrode can easily light a “NENU” panel composed of 51 LED bulbs (Figure S6).

### 3.2. Analysis and Discussions

The electrochemical performances of lithium–sulfur batteries with different biomass host materials are summarized in Table S4. The WPC-Mo_2_C exhibits strong cycle stability at 1 C. Meanwhile, the WPC-Mo_2_C shows a higher specific capacity under high sulfur loading than the reported results. These results directly reflect that waste pinecone-derived carbon modified with Mo_2_C nanoparticles has excellent properties as the sulfur host. First, the waste pinecone-derived carbon has excellent structural stability, and the Mo_2_C nanoparticle modification can significantly improve the electronic conductivity of the WPC-Mo_2_C, so as to improve the discharge rate by offering a conductive path and an effective sulfur fixation ability. In addition, the WPC-Mo_2_C has a strong chemical adsorption capacity and catalytic performance, and it can effectively adsorb polysulfide, inhibit the shuttle effect, and accelerate the conversion of sulfur species, thus improving the overall efficiency of Li-S batteries.

## 4. Conclusions

In summary, Mo_2_C nanoparticle-modified, pinecone-derived carbon was used as a host for lithium–sulfur batteries. After introducing Mo_2_C nanoparticles to decorate the carbon base, the WPC-Mo_2_C composite demonstrated excellent electrochemical performance, which can be attributed to the following reasons: (1) The large specific surface area of the WPC-Mo_2_C provides sufficient space to accommodate the volume expansion of sulfur with a high sulfur loading, further improving the specific capacity of lithium–sulfur batteries at 5.5 mg cm^−2^. (2) The non-metal element doping of the pinecones, such as with N and O, changes the structure of the carbon materials and realizes the adsorption of LiPSs, enhancing the interface compatibility between the electrolyte and the electrode and increasing the diffusion rate and reaction rate of ions in Li-S batteries. (3) As a polar material, the loading of Mo_2_C nanoparticles can effectively adsorb LiPSs chemically to limit the shuttle effect and catalyze so as to accelerate the reaction process of sulfur redox conversion, improving the charge and discharge efficiency and enhancing the rate performance of Li-S batteries. As a result, the WPC-Mo_2_C/S electrode exhibits a cycle performance with a capacity decay rate of 0.074% per cycle after 600 cycles at 1 C. Even at a high sulfur loading of 5.5 mg cm^−2^, the specific capacity of the WPC-Mo_2_C/S electrode can reach 5.1 mAh cm^−2^ after 60 cycles at 0.2 C. This work emphasizes the application of waste biomass materials in lithium–sulfur batteries and provides a means for the design and development of low-cost, environmentally friendly, and efficient lithium–sulfur batteries.

## Data Availability

The original contributions presented in this study are included in the article/Supplementary Materials. Further inquiries can be directed to the corresponding author.

## Supplementary Materials

The following supporting information can be downloaded at: https://www.mdpi.com/article/10.3390/ma18051141/s1, Figure S1: N_2_ adsorption/desorption isotherms and the corresponding pore size distributions of WPC-Mo_2_C; Figure S2: N_2_ adsorption/desorption isotherms and the corresponding pore size distributions of WPC; Figure S3: The XPS full spectrum of WPC-Mo_2_C; Figure S4: GITT profile of WPC; Figure S5: CV curves of WPC at a scan rate of 0.1–0.5 mV s^−1^; Figure S6: Digital photograph of LED device lit by Li-S battery based on the WPC-Mo_2_C electrode. Table S1: Mo_2_C content of WPC-Mo_2_C detected by ICP-ONES; Table S2: Li^+^ diffusion coefficients of the cells with different cathodes; Table S3: Summary of *R_s_* and *R_ct_* values for cells with different cathodes; Table S4: Comparison of the electrochemical performances of the lithium sulfur battery with different biomass host materials. References [22,32,33,34,35,36] are cited in the supplementary materials.
