# Supplementary material for: Transforming Waste into Valuable Resources: Mo2C Nanoparticles Modified Waste Pinecone-Derived Carbon as an Effective Sulfur Host for Lithium–Sulfur Batteries
Source: Materials (Basel). 2025 Mar 4;18(5):1141. doi: 10.3390/ma18051141 (PMC11902224; doi:10.3390/ma18051141)
Supplement: Supplementary file 1 [file materials-18-01141-s001.zip › materials-3505441-supplementary.pdf]

Supporting information

Transforming Waste into Valuable Resources: Mo_2C Nanoparticles Modified Waste Pinecone-Derived Carbon as an Effective Sulfur Host for Lithium-Sulfur Batteries

Zhe Yang[1], Yicheng Han[1], Kai Chen[2], Guodong Zhang[2], Shuangxi Xing[1*]*

[1.] Faculty of Chemistry, Northeast Normal University; Changchun, 130024, China.

[2.] Department of Physics, Research Institute for Biomimetics and Soft Matter, Fujian Provincial Key Laboratory for Soft Functional Materials, Xiamen University, Xiamen 361005, China.

***Corresponding Author**

Guodong Zhang, E−mail: zhanggd710@nenu.edu.cn (G. Zhang)

Shuangxi Xing, E−mail: xingsx737@nenu.edu.cn (S. Xing)

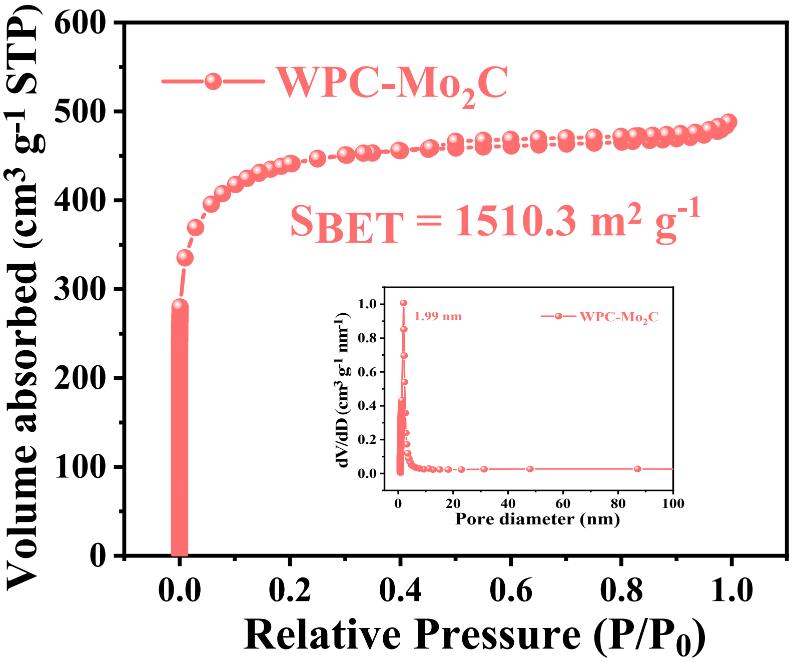

Figure S1. N_2 adsorption/desorption isotherms and the corresponding pore size distributions of WPC-Mo_2C.

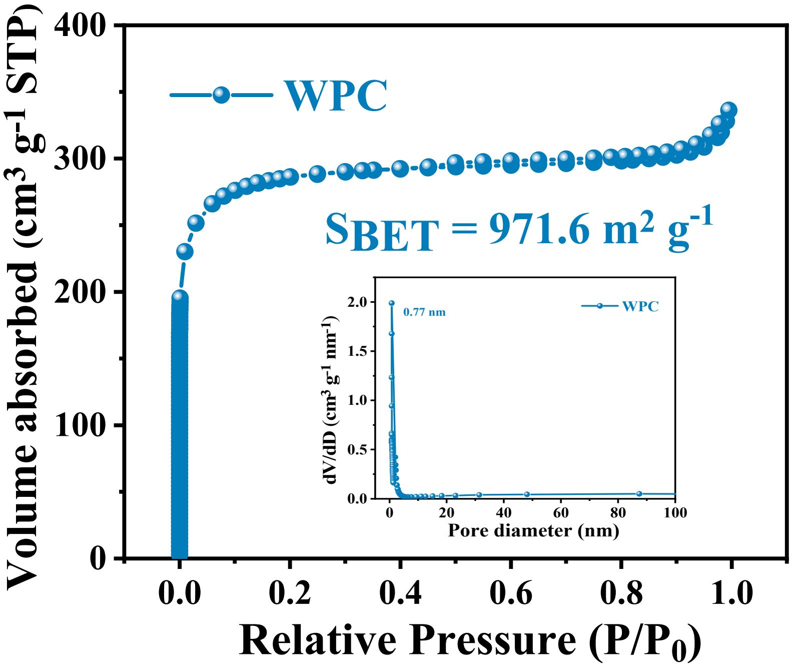

Figure S2. N_2 adsorption/desorption isotherms and the corresponding pore size distributions of WPC.

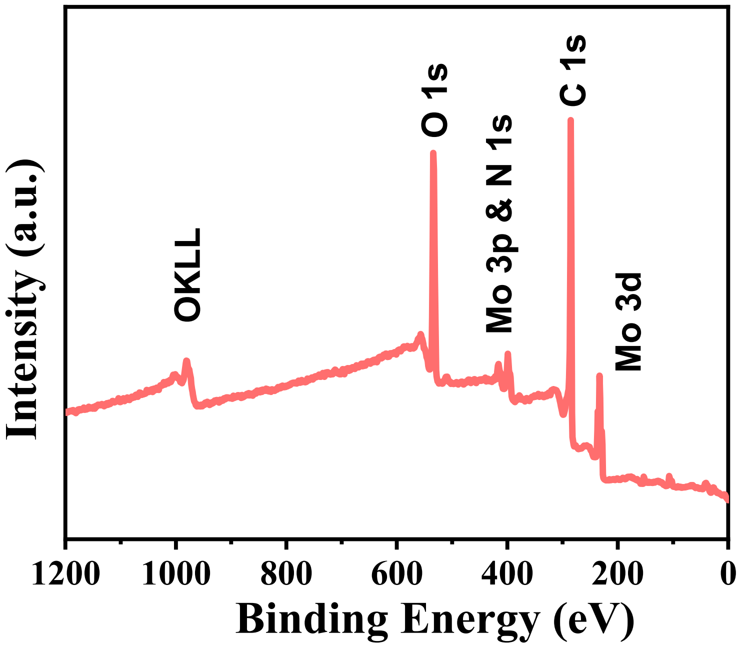

Figure S3. The XPS full spectrum of WPC-Mo_2C.

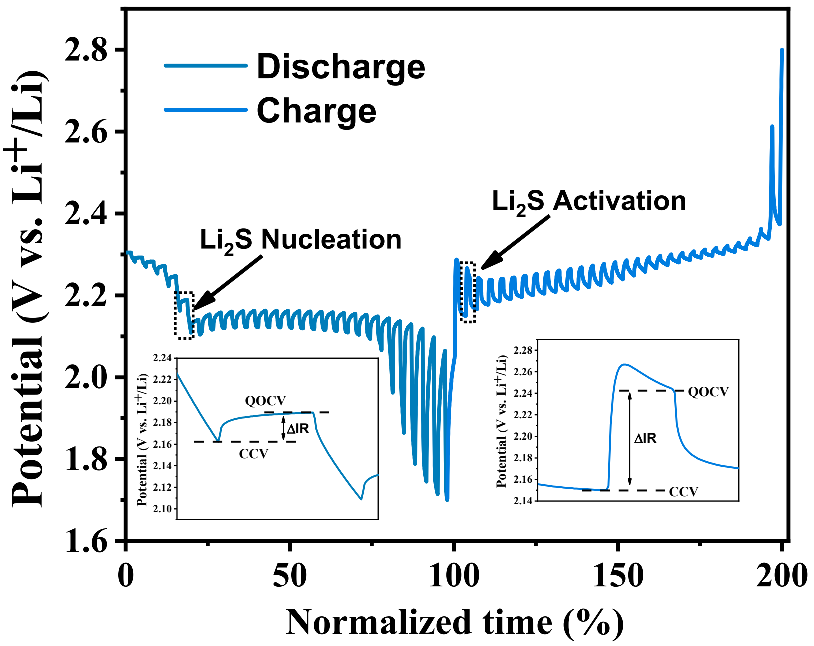

Figure S4. GITT profile of WPC.

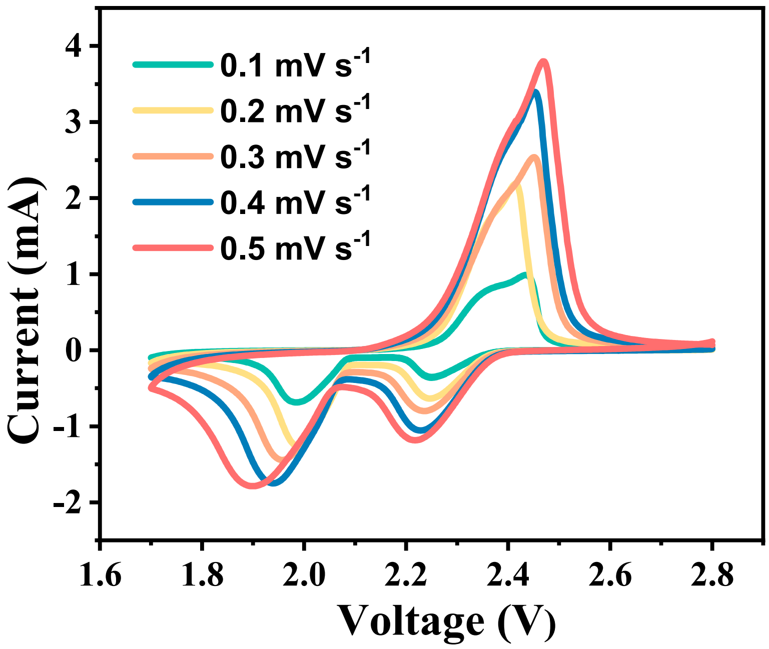

Figure S5. CV curves of WPC at a scan rate of 0.1-0.5 mV s^{-1}.

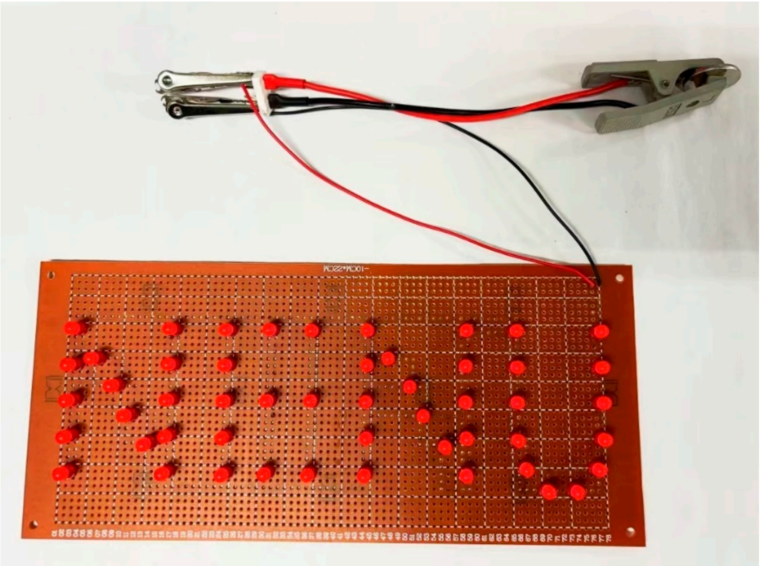

Figure S6. Digital photograph of LED device lit by Li-S battery based on the WPC-Mo_2C electrode.

Table S1. Mo_2C content of WPC-Mo_2C detected by ICP-ONES

Sample	Units (μg/mL)	Mo_2C content (wt%)
WPC-Mo_2C	2.264	0.91

Table S2. Li^+ diffusion coefficients of the cells with different cathodes

Cathodes	D_{Li^+} at peak A ($cm^2\ s^{-1}$)	D_{Li^+} at peak B ($cm^2\ s^{-1}$)	D_{Li^+} at peak C ($cm^2\ s^{-1}$)
WPC-Mo_2C	4.98×10^{-9}	4.69×10^{-9}	4.52×10^{-8}
WPC	2.59×10^{-9}	4.34×10^{-9}	2.72×10^{-8}

Table S3. Summary of R_s and R_{ct} values for cells with different cathodes

Cathodes	R_s (Ω)	R_{ct} (Ω)
WPC-Mo_2C	2.792	110
fitting	2.79	111
WPC	4.729	177.9
fitting	4.73	178

Table S4. Comparison of the electrochemical performances of the lithium sulfur battery with different biomass host materials

Host biomass materials	Sulfur loading (mg cm^{-2})	Cycles	Current density (C)	Initial capacity (mA h g^{-1})	Reversible capacity (mA h g^{-1})	Fading rate per cycle (%)	Ref.
pinecones	1.2	600	1	843.3	467.5	0.074	This work
	5.5	60	0.2	1042.2	918.5	0.19	
rice straws	5.28	90	0.1	779	583	0.28	[1]
rice husk	4	100	0.2	1035	603	0.42	[2]
Ganoderma Lucidum	1	300	0.5	872	631	0.092	[3]
lignocellulose	1.2	300	1	825	582	0.098	[4]
Meringue	1	100	0.3	1100	800	0.27	[5]
catkins	1	500	0.5	700	542.5	0.043	[6]

[1] J. Wang, L. Wu, L. Shen, Q. Zhou, Y. Chen, J. Wu, Y. Wen, J. Zheng, CoO embedded porous biomass-derived carbon as dual-functional host material for lithium-sulfur batteries, Journal of Colloid and Interface Science 640 (2023) 415-422. https://doi.org/https://doi.org/10.1016/j.jcis.2023.02.123.
[2] F. Sultanov, N. Zhumasheva, A. Dangaliyeva, A. Zhaisanova, N. Baikalov, B. Tatykayev, M. Yeleuov, Z. Bakenov, A. Mentbayeva, Enhancing lithium-sulfur battery performance with biomass-derived graphene-like porous carbon and NiO nanoparticles composites, Journal of Power Sources 593 (2024) 233959. https://doi.org/https://doi.org/10.1016/j.jpowsour.2023.233959.
[3] J. Cui, J. Liu, X. Chen, J. Meng, S. Wei, T. Wu, Y. Wang, Y. Xie, C. Lu, X. Zhang, Ganoderma Lucidum-derived erythrocyte-like sustainable materials, Carbon 196 (2022) 70-77. https://doi.org/https://doi.org/10.1016/j.carbon.2022.04.034.
[4] J. Xu, P. Zhou, L. Dai, Y. Gui, L. Yuan, X. Shen, C. Zhang, K. Huo, A scalable waste-free biorefinery inspires revenue from holistic lignocellulose valorization, Green Chemistry 23(16) (2021) 6008-6019. https://doi.org/10.1039/D1GC01720A..
[5] J.R. Choi, E. Kim, B.-I. Park, I. Choi, B.-H. Park, S.-B. Lee, J.H. Lee, S. Yu, Meringue-derived hierarchically porous carbon as an efficient polysulfide regulator for lithium-sulfur batteries, Journal of Industrial and Engineering Chemistry 115 (2022) 355-364. https://doi.org/https://doi.org/10.1016/j.jiec.2022.08.019.
[6] L. Fan, Z. Li, W. Kang, B. Cheng, Biomass-derived tube-like nitrogen and oxygen dual-doped porous carbon in the sulfur cathode for lithium sulfur battery, Renewable Energy 155 (2020) 309-316. https://doi.org/https://doi.org/10.1016/j.renene.2020.03.153.
